# An evolutionarily conserved glycine-tyrosine motif forms a folding core in outer membrane proteins

**DOI:** 10.1371/journal.pone.0182016

**Published:** 2017-08-03

**Authors:** Marcin Michalik, Marcella Orwick-Rydmark, Michael Habeck, Vikram Alva, Thomas Arnold, Dirk Linke

**Affiliations:** 1 Department of Biosciences, University of Oslo, Oslo, Norway; 2 Previous affiliation: Department of Protein Evolution, Max Planck Institute for Developmental Biology, Tübingen, Germany; 3 Statistical inverse problems in Biophysics, Max Planck Institute for Biophysical Chemistry, Göttingen, Germany; 4 Felix Bernstein Institute for Mathematical Statistics in the Biosciences, University of Göttingen, Göttingen, Germany; 5 Department of Protein Evolution, Max Planck Institute for Developmental Biology, Tübingen, Germany; 6 Boehringer Ingelheim Veterinary Research Center, Hannover, Germany; Russian Academy of Medical Sciences, RUSSIAN FEDERATION

## Abstract

An intimate interaction between a pair of amino acids, a tyrosine and glycine on neighboring β-strands, has been previously reported to be important for the structural stability of autotransporters. Here, we show that the conservation of this interacting pair extends to nearly all major families of outer membrane β-barrel proteins, which are thought to have originated through duplication events involving an ancestral ββ hairpin. We analyzed the function of this motif using the prototypical outer membrane protein OmpX. Stopped-flow fluorescence shows that two folding processes occur in the millisecond time regime, the rates of which are reduced in the tyrosine mutant. Folding assays further demonstrate a reduction in the yield of folded protein for the mutant compared to the wild-type, as well as a reduction in thermal stability. Taken together, our data support the idea of an evolutionarily conserved ‘folding core’ that affects the folding, membrane insertion, and thermal stability of outer membrane protein β-barrels.

## Introduction

Outer membranes (OM) of Gram-negative bacteria and of mitochondria and chloroplasts that evolved from prokaryotic organisms [1,2] mainly contain transmembrane proteins with a β-barrel structure, called outer membrane proteins (OMPs). Such β-barrel membrane proteins are not found in other membrane systems, presumably because they form a cylindrical pore that allows for the passage of ions by diffusion even in the smallest of examples [3–5]. Known β-barrel structure sizes range from 8 to 26 β-strands, and the strand number–in prokaryotes at least–is always a multiple of 2, with both N- and C-termini residing in the periplasm. Such OMPs have very diverse functions, including active and passive transport of nutrients and protein export (as part of various secretion systems), and are classified as enzymes, receptors, structural proteins, or adhesins [6]. Their specific function is often based on their pore structure, or on their additional periplasmic or surface-exposed domains.

All OMPs are evolutionarily related and presumably evolved by duplication events of β-hairpin repeats [4,7]. This evolutionary relationship and sequence features, resulting from functional as well as structural constraints, have been exploited for the prediction of OMP β-barrels from sequence alone. There are two main features: an alternating pattern of hydrophilic and hydrophobic residues indicative of transmembrane β-strands, and a band of aromatic residues (tryptophan, phenylalanine and tyrosine) which faces the lipid-water-interface region in both leaflets of the membrane [8].

Membrane insertion and folding of OMPs *in vivo* is a much-studied but poorly understood process. OMPs, like all other bacterial proteins, are synthesized by ribosomes in the cytoplasm. Their sequences include an N-terminal signal peptide that is recognized by the Sec translocation system which transports the unfolded chain through the inner membrane (IM) [9,10]. After the signal peptide has been cleaved by the signal peptidase, the protein is bound by periplasmic chaperones (*SurA*, *Skp*, *DegP*), to protect the unfolded state against aggregation, misfolding, and proteolytic cleavage [11]. As there is no external source of energy available in the periplasm in the form of ATP or a proton gradient, the process of trafficking an OMP across the periplasm must be exergonic, and the energy may come from the folding of transported products [12]. The folding of OMPs into the OM is mediated by the β-barrel-assembly machinery (BAM) complex, which *in E*. *coli* consists of the BamA protein (itself an OMP) and four additional lipoproteins. The exact mechanism by which OMPs are inserted and folded into the OM remains unclear, although high-resolution structures and many *in vitro* and *in vivo* studies are available [13–16]. The most popular hypothesis currently suggests that the BAM complex initially recognizes the unfolded chaperone-bound OMP, which is subsequently threaded through the pore of BamA where insertion of β-strands into the membrane occurs through the lateral opening of BamA strands 1 and 16 [14,17,18]. It is worth noting however, that at least small OMPs are able to spontaneously insert into lipid membranes *in vitro* without help by any other factors [19], suggesting that the BAM complex only facilitates a process whose driving force is already encoded into the OMP sequence.

Beyond their general sequence features as described above, OMPs include specific sequence motifs for their function, *i*.*e*. a C-terminal motif that plays a role in BAM recognition [20,21]. In autotransporters, another recently recognized motif includes a conserved glycine and tyrosine pair, where the glycine can also be substituted for a threonine, and the tyrosine for a phenylalanine or tryptophan. This pair is present on neighboring β-strands and forms a clamp inside the barrel. This motif, described as a mortise-tenon motif, has been suggested to provide structural stability to autotransporters by aligning and locking the neighboring β-strands [22]. A similarly conserved tyrosine-glycine pair had been found earlier in alignments of 8-stranded OMPs [23], but its possible function in protein structure or folding was not discussed further.

**Fig 1:**
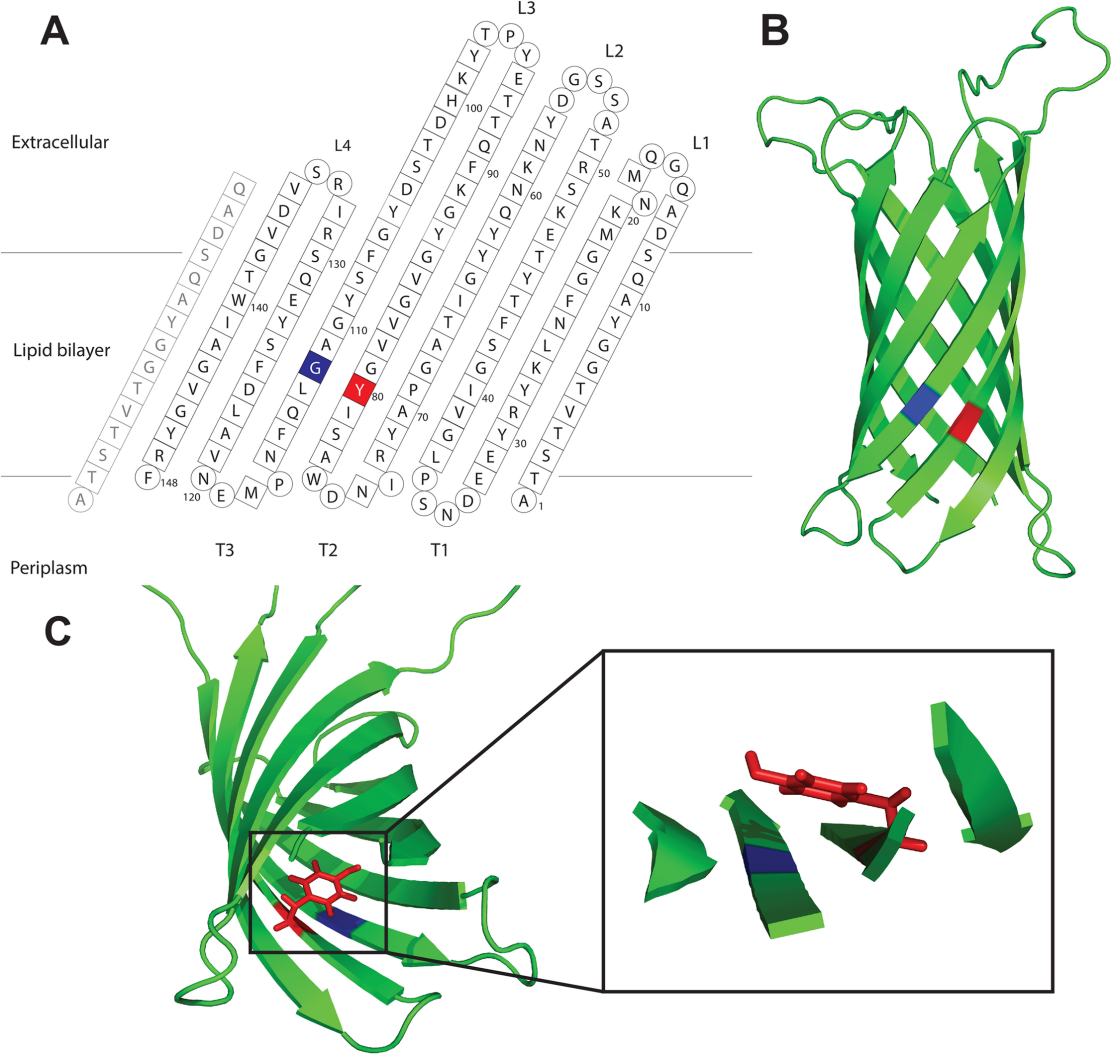
OmpX structure. **(A)** The topology model of OmpX with the highlighted mortise-tenon motif in red (Y80) and blue (G112) on the 5^th^ and 6^th^ β-strands. Residues forming the β-strands are represented as squares, while loop residues are shown as circles (adapted from [24]). **(B)** The OmpX solution NMR structure in 3D representation using PyMOL [PDB code 2MNH]. **(C)** The mortise-tenon motif involves residues Y80 (red) and G112 (blue). The tyrosine’s aromatic side chain is positioned in a planar manner over the glycine in the neighboring strand (G112).

Using bioinformatics tools, we assessed the evolutionary conservation of this motif amongst OMPs. Further, to understand its functional role, we used a combination of mutagenesis and a variety of biophysical tools to study the folding kinetics and structural stability of the prototypical 8-stranded β-barrel bacterial OMP, OmpX [24]. The tyrosine-glycine motif in OmpX involves residues Y80 and G112 (Fig 1). The aromatic ring of Y80 is oriented towards the lumen of the barrel and is located over G112 on the neighboring strand, the small size of which allows for Y80 to position in a planar manner directly over it (Fig 1), forming an apparent mortise-tenon motif analogous to that described in autotransporters [22]. The biological function of OmpX is not fully understood, but it is believed to play a role in neutralizing host defense mechanisms [24]. Previous studies have shown that it is a very robust protein that can still fold *in vivo* and *in vitro* after the introduction of mutations [4,25] and that it can easily be refolded from inclusion bodies into detergent micelles or liposomes [26,27].

## Results

### Bioinformatics

To investigate how widespread mortise-tenon motifs are across all OMPs, we decided to search for them in a comprehensive set representative of all known OMP types. For this, we chose the Structural Classification of Proteins (SCOPe) database [28], which classifies proteins of known structure into hierarchies based on evolutionary and structural criteria. SCOPe classifies OMPs into 7 superfamilies, comprising a total of 15 families. Using sequence and structural analysis, we searched for mortise-tenon motifs in these 15 families, as described in the Methods section. We detected highly conserved mortise-tenon motifs in 13 of these families (Table 1), showing that they not restricted to only autotransporters and eight-stranded barrels, but that they are in fact more widespread among OMPs. We analyzed these results in more detail for the three major classes of OMPs with multiple exemplars of known structure: the 8-stranded, 16-stranded, and 22-stranded OMPs.

**Table 1:** Summary of results into the conservation of the mortise-tenon motif across all families of OMPs as classified by the SCOPe database. The mortise-tenon motif is found to be conserved in 13 of the 15 OMP families.

SCOPe ID	SCOP Family	PFAM ID	PFAM Family	Strand-number	Mortise‐Tenon motif location
f.4.1.1	Outer membrane protein	PF01389	OmpA_membrane	8	OmpA; 1QJP; Y94 –G126
f.4.1.2	Outer membrane enzyme PagP	PF07017	PagP	8	PagL; 2ERV; F78 –G111
f.4.1.3	GNA1870 immunodominant domain-like	PF01298	TbpB_B_D	8	no widely conserved motifs
f.4.1.4	PsbO-like	PF01716	MSP	8	PsbO; 3WU2, chain o; F146—G195
f.4.2.1	Outer membrane phospholipase A (OMPLA)	PF02253	PLA1	12	OMPLA; 1QD5; G139 –Y159,
f.4.3.1	Porin	PF00267	Porin_1	16	OmpK36; 1OSM; Y14 –G44, Y58 –G87, Y310 –G335
f.4.3.2	Maltoporin-like	PF02264	LamB	18	LamB; 1AF6; F366 –G414, W101 –G128
f.4.3.3	Ligand-gated protein channel	PF00593	TonB_dep_Rec	22	ButB; 1NQE; G314 –Y341; Y436 –G463
f.4.3.4	Outer membrane protein transport protein	PF03349	Toluene_X	14	none
f.4.4.1	Outer membrane protease OMPT	PF01278	Omptin	10	OMPT; 1I78; G133 –Y180, Y248 –G292
f.4.4.2	Outer membrane adhesin/invasin OpcA	PF07239	OpcA	10	OpcA; 2VDF; Y154 –G198, W158—G194, F212 –G246
f.4.5.1	Autotransporter	PF03797	Autotransporter	12	EspP; 2QOM; G1081 –Y1108
f.4.6.1	Tsx-like channel	PF03502	Channel_Tsx	12	tsx; 1TLW; G126 –Y144
f.4.7.1	OccD-like	PF03573	OprD	18	OpdC; 3SY9; G159 –W193, G195 –Y209
f.4.7.2	OccK-like	PF03573	OprD	18	OpdF; 3SZD; G153 –F184, G186 –W201

**Fig 2:**
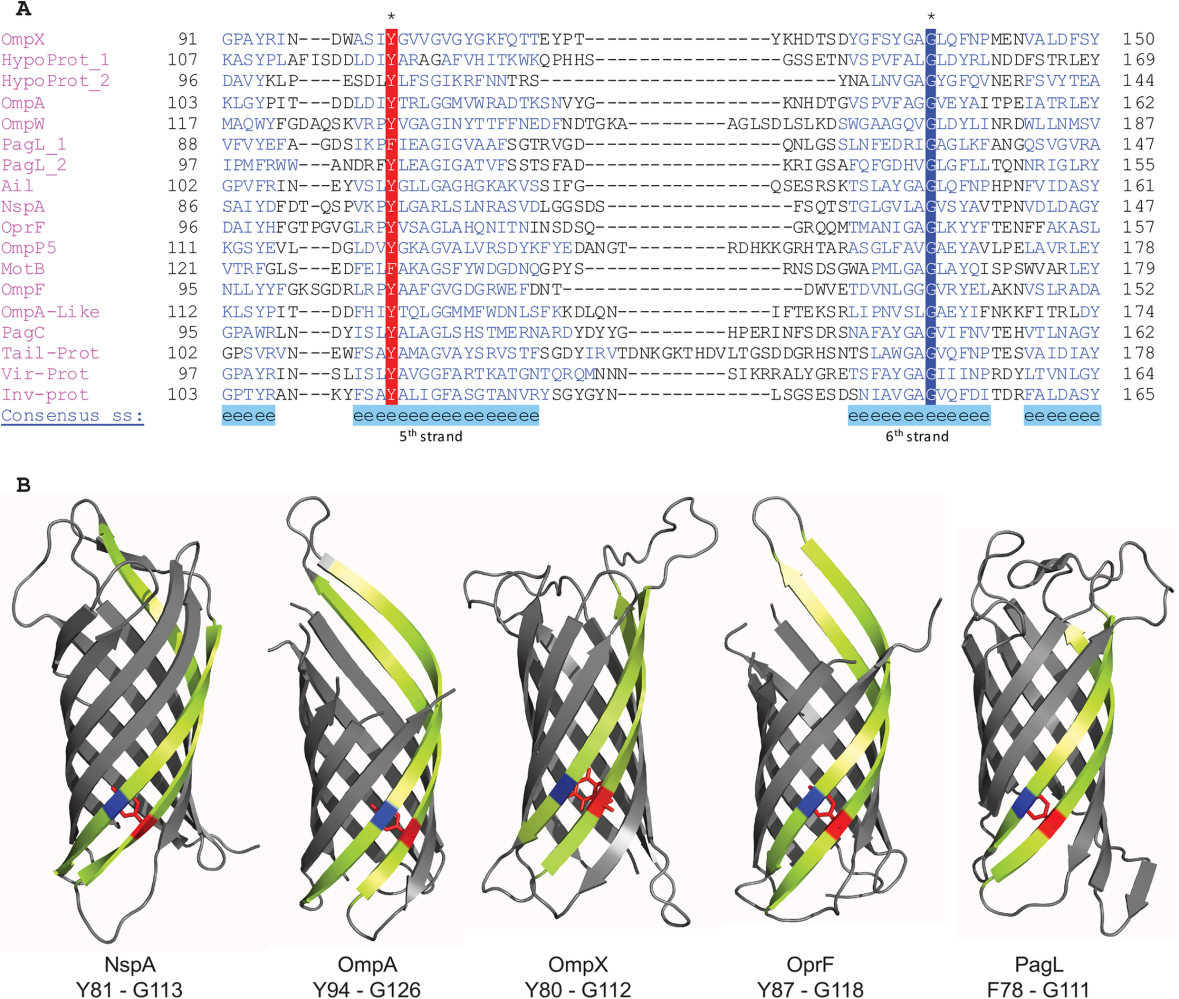
**(A)** Alignments of the mortise-tenon regions in 8-stranded OMP β-barrels. There is 100% conservation of the glycine (dark blue, strand 6^th^) and very high conservation of the tyrosine (red, strand 5^th^) in the mortise-tenon motif. Alignments were prepared using PROMALS3D web server; the predicted β-strands are colored in blue, and the consensus secondary structure prediction is given below the alignment. **OmpX**—APC51088.1 Outer membrane protein X [*Escherichia coli* str. K-12 substr. W3110]; **HypoProt_1**—WP_039105847.1 hypothetical protein [*Frischella perrara*]; **HypoProt_2**—WP_044834085.1 hypothetical protein [*Thalassomonas actiniarum*]; **OmpA**—BAA35715.1 Outer membrane protein A [*Escherichia coli* str. K-12 substr. W3110]; **OmpW**—CDO14140.1 Outer membrane protein W [*Klebsiella pneumoniae*]; **PagL_1**—BAR70092.1 PagL [*Pseudomonas aeruginosa*]; **PagL_2**—AGW82166.1 PagL [*Bordetella avium*]; **Ail**—AAB36601.1 Ail [*Yersinia pseudotuberculosis*]; **NspA**—CAX50548.1 NspA [*Neisseria meningitidis* 8013]; **OprF**—AFM37279.1 OprF [*Pseudomonas aeruginosa*]; **OmpP5**—AAA03346.1 outer membrane protein P5 [*Haemophilus influenzae*]; **MotB**—WP_012154185.1 flagellar motor protein MotB [*Shewanella pealeana*]; **OmpF**—WP_013260126.1 Outer membrane porin F [*gamma proteobacterium* HdN1]; **OmpA**-Like—WP_011053841.1 OmpA-like protein [*Buchnera aphidicola*]; **PagC**—WP_067708083.1 PagC [*Erwinia* sp. ErVv1]; **TailProt**—CSH02213.1 Tail assembly protein [*Shigella sonnei*]; **VirProt**—WP_038626525.1 virulence protein [*Pantoea* sp. PSNIH2]; **InvProt**—SDG65145.1 attachment invasion locus protein [*Vibrio xiamenensis*]. **(B)** Structures of different 8-stranded β barrel proteins. The 5^th^ and 6^th^ β-strands are highlighted in yellow and the mortise-tenon motif is highlighted in blue and red accordingly. PDB codes: NspA [1P4T]; OmpA[1QJP]; OmpX[2MNH]; OprF[4RLC]; PagL[2ERV].

**Fig 3:**
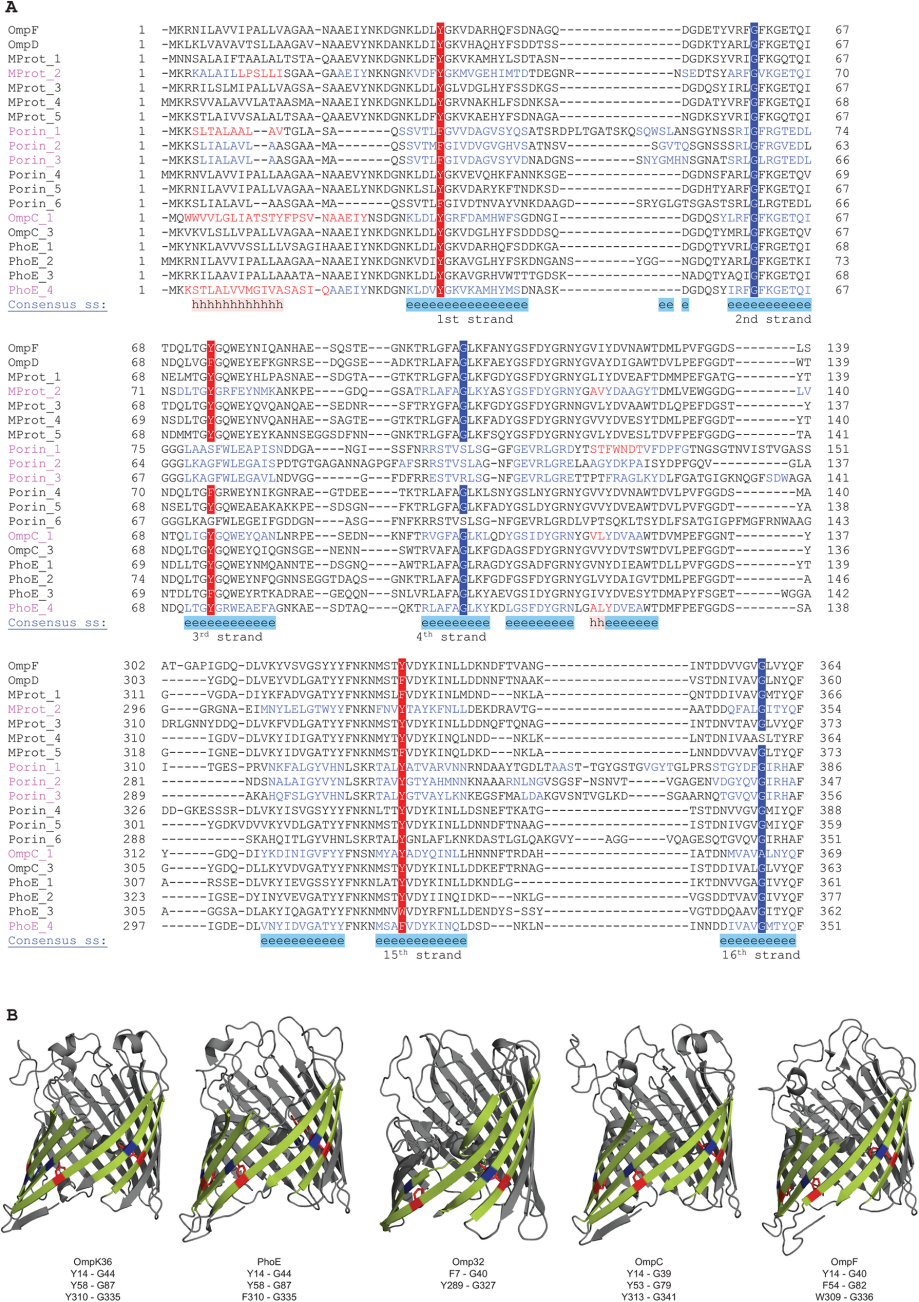
**(A)** Alignments of the mortise-tenon motifs in 16-stranded OMP β-barrels. Three conserved motifs are present on the 1^st^ and 2^nd^ β-strands, 3^rd^ and 4^th^ β-strands and on the 15^th^ and 16^th^ β-strands. The color scheme is the same as in Fig 2. **OmpF**—AJW81160.1 OmpF [*Yersinia ruckeri*]; **OmpD**—GAL44027.1 outer membrane porin OmpD [*Citrobacter werkmanii* NBRC 105721]; **MProt_1**—WP_052899673.1 membrane protein [*Erwinia iniecta*]; **MProt_2**—WP_045859316.1 membrane protein [*Raoultella terrigena*]; **MProt_3**—WP_034791683.1 membrane protein [*Ewingella americana*]; **MProt_4**—WP_024486820.1 membrane protein [*Serratia fonticola*]; **MProt_5**—WP_028724068.1 membrane protein [*Pantoea ananatis*]; **Porin_1**—SCX53975.1 Outer membrane protein (porin) [*Variovorax sp*. EL159]; **Porin_2**—WP_005794534.1 porin [*Acidovorax delafieldii*]; **Porin_3**—WP_003058791.1 porin [*Comamonas testosteroni*]; **Porin_4**—WP_011146030.1 porin [*Photorhabdus luminescens*]; **Porin_5**—WP_035609637.1 porin [*Edwardsiella ictaluri*]; **Porin_6**—WP_034397082.1 porin [*Delftia acidovorans*]; **OmpC_1**—WP_019081949.1 porin OmpC [*Yersinia enterocolitica*]; **OmpC_3**—WP_058683256.1 porin OmpC [*Enterobacter cloacae*]; **PhoE_1**—WP_002439127.1 phosphoporin PhoE [*Shimwellia blattae*]; **PhoE_2**—WP_017456205.1 phosphoporin PhoE [*Kosakonia sacchari*]; **PhoE_3**—WP_072077160.1 phosphoporin PhoE [*Salmonella enterica*]; **PhoE_4**—WP_032085013.1 phosphoporin PhoE [*Escherichia coli*]. **(B)** Structures of different 16-stranded β barrel proteins. The 1^st^-2^nd^, 3^rd^-4^th^ and 15^th^-16^th^ β-strands are highlighted in yellow and the mortise-tenon motifs are highlighted in blue and red accordingly. The amino-acid pairs forming the motif are given under the names of the proteins. PDB codes: OmpK36 [1OSM]; PhoE [1PHO]; Omp32 [2FGQ]; OmpC [2J1N]; OmpF [3NSG].

**Fig 4:**
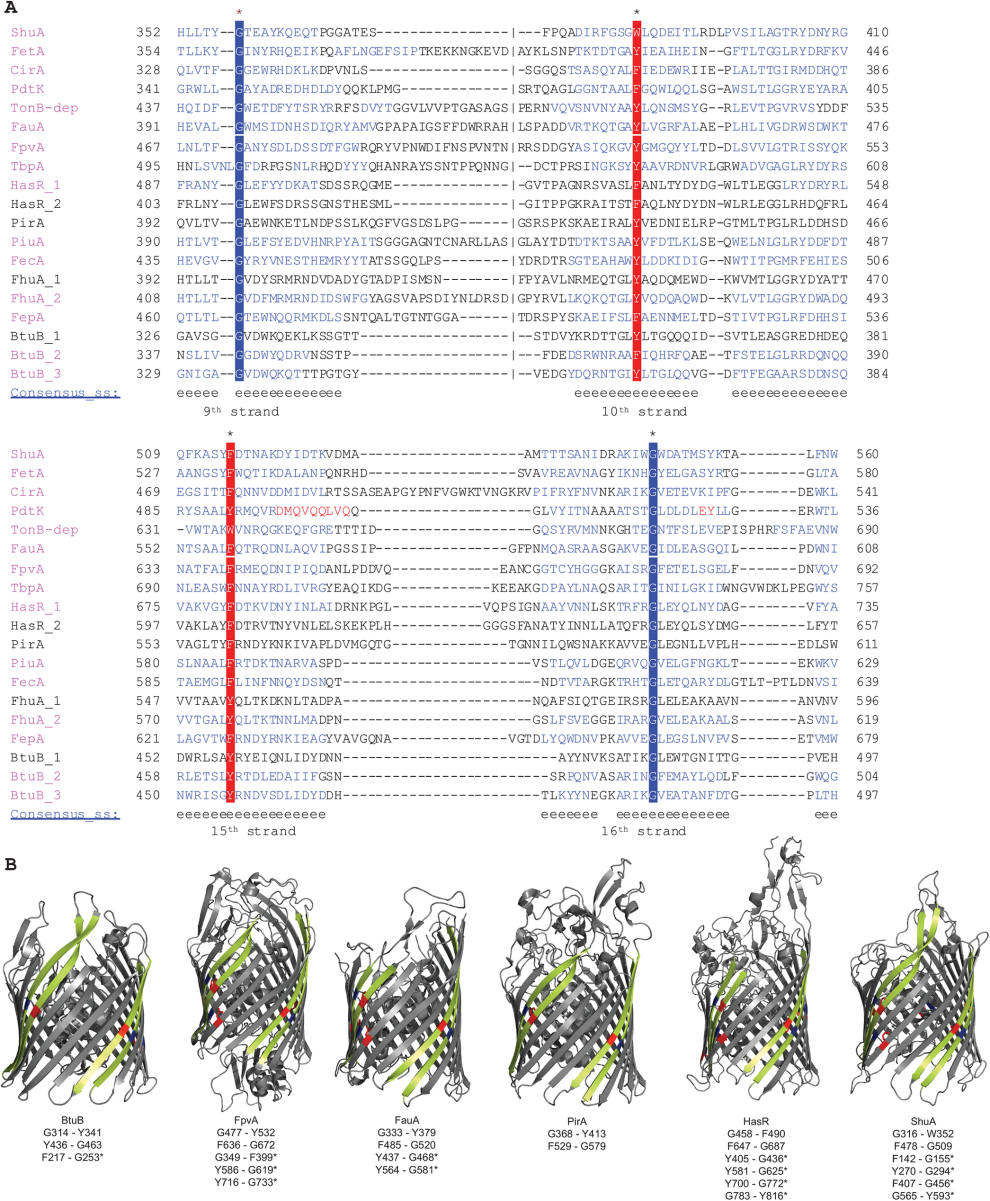
**(A)** Alignments of the mortise-tenon motifs in 22-stranded OMP β-barrels. Two conserved motifs are present on the 9^th^ and 10^th^ β-strands and on the 15^th^ and 16^th^ β-strands. Compared to 8-stranded proteins, the glycine in the motif is also completely conserved, while the tyrosine in the motif is less conserved, with the aromatic residues phenylalanine and tryptophan also present at a higher frequency. The color scheme is the same as in Fig 2. **ShuA**—AIN19718.1 outer membrane heme receptor ShuA [*Yersinia kristensenii*]; **FetA**—AHW74795.1 FetA [*Neisseria meningitidis*]; **CirA**—CDO15561.1 cirA [*Klebsiella pneumoniae*]; **PdtK**—ABC68350.1 PdtK [*Pseudomonas putida*]; **TonB-dep**—WP_043878013.1 TonB-dependent receptor [*Pectobacterium atrosepticum*]; **FauA**—AAD26430.1 ferric alcaligin siderophore receptor FauA [*Bordetella pertussis*]; **FpvA**—AOX26958.1 FpvA [*Pseudomonas aeruginosa*]; **TbpA**—AAF81744.1 transferrin-binding protein A [*Neisseria meningitidis*]; **HasR_1**—CAE46936.1 hasR [*Serratia marcescens*]; **HasR_2**—AIN17416.1 hasR protein [*Yersinia kristensenii*]; **PirA**—CFU88752.1 ferric enterobactin receptor PirA [*Pseudomonas aeruginosa*]; **PiuA**—SCM64637.1 PiuA putative outer membrane ferric siderophore receptor [*Pseudomonas aeruginosa*]; **FecA**—AAL08456.2 FecA [*Shigella flexneri* 2a]; **FhuA_1**—CDO15921.1 fhuA [*Klebsiella pneumoniae*]; **FhuA_2**—ANK05474.1 fhuA [*Escherichia coli* O25b:H4]; **FepA**—ADB98042.1 FepA [*Escherichia coli*]; **BtuB_1**—CDO16333.1 btuB [*Klebsiella pneumoniae*]; **BtuB_2**—AEV65209.1 BtuB [*Pseudomonas fluorescens* F113]; **BtuB_3**—EFE98543.1 btuB [*Escherichia coli* FVEC1412]. **(B)** Structures of different 22-stranded OMP β-barrels. The 9^th^-10^th^ and 15^th^-16^th^ β-strands are highlighted in yellow and the mortise-tenon motifs are highlighted in blue and red accordingly. The amino acid pairs forming the motif are given under the names of the proteins. Observed mortise-tenon pairs that were at non-conserved positions are marked with an astrix. PDB codes: BtuB [1NQE]; FpvA [2O5P]: FauA [3EFM]; PirA [5FR8]; HasR [3CSN]; ShuA [3FHH].

For 8-stranded β-barrels, which are grouped into a superfamily (SCOPe f.4.1) with 4 families in SCOPe, there is one motif located on the 5^th^ and 6^th^ β-strand which in most cases consists of a glycine-tyrosine pair (Fig 2). While this motif is highly conserved in three of these families, one family (SCOPe f.4.1.3) contains no widely conserved motifs. In 16-stranded β-barrels (SCOPe family f.4.3.1), we found multiple mortise-tenon pairs, with the most conserved pairs located on strands 1 and 2, 3 and 4, and 15 and 16 (Fig 3). Here, the aromatic residue is somewhat less conserved compared to 8-stranded β-barrels, with more examples of phenylalanine and tryptophan replacing tyrosine (Fig 3A). Finally, in 22-stranded-β- barrels (SCOPe family f.4.3.3), we also found multiple mortise-tenon pairs, with the most conserved ones located between β-strands 9 and 10 and 15 and 16 (**Fig 4**). Similar to the 16-β-stranded barrels, the tyrosine can be substituted for a phenylalanine or tryptophan (**Fig 4A**).

The presence of this motif in most families of OMP β–barrels and its extreme positional conservation strongly suggests that it is evolutionarily old, and presumably has a very basic function that is independent of the different transport or enzymatic functions of this broad family of proteins. Our previous studies on OMP evolution using OmpX as a model system showed that OmpX can fold in almost all permutations of added or deleted β-hairpins, as long as the central hairpin is kept [4]. The fact that this hairpin contains the conserved tyrosine residue described here led us to the hypothesis that the mortise-tenon pair presents a conserved ‘folding core’ for β-barrel proteins.

### Folding kinetics

To evaluate the role of the conserved mortise-tenon motif in OMP folding, stopped-flow fluorescence experiments to measure folding rates were carried out on wt OmpX as well as a mutant in which Y80 was mutated to a glycine to remove the aromatic side chain completely (Y80G). OmpX contains two tryptophan residues at positions 76 and 140, and the refolding kinetics were monitored using the change in fluorescence intensity after folding, using a fluorescence detector with an excitation wavelength of 295 nm to avoid contribution from other aromatic residues, and a cutoff filter at 320 nm to capture maximal fluorescence increase without contributions of scattered excitation light. Measuring tryptophan fluorescence intensity over time is very useful in measuring kinetics of OMPs: tryptophan is sensitive to the polarity of the local environment, and during protein folding shows a characteristic red-shift in its emission spectrum. In the case of OmpX this is coupled to an increase in the fluorescence intensity [29–31].

**Fig 5:**
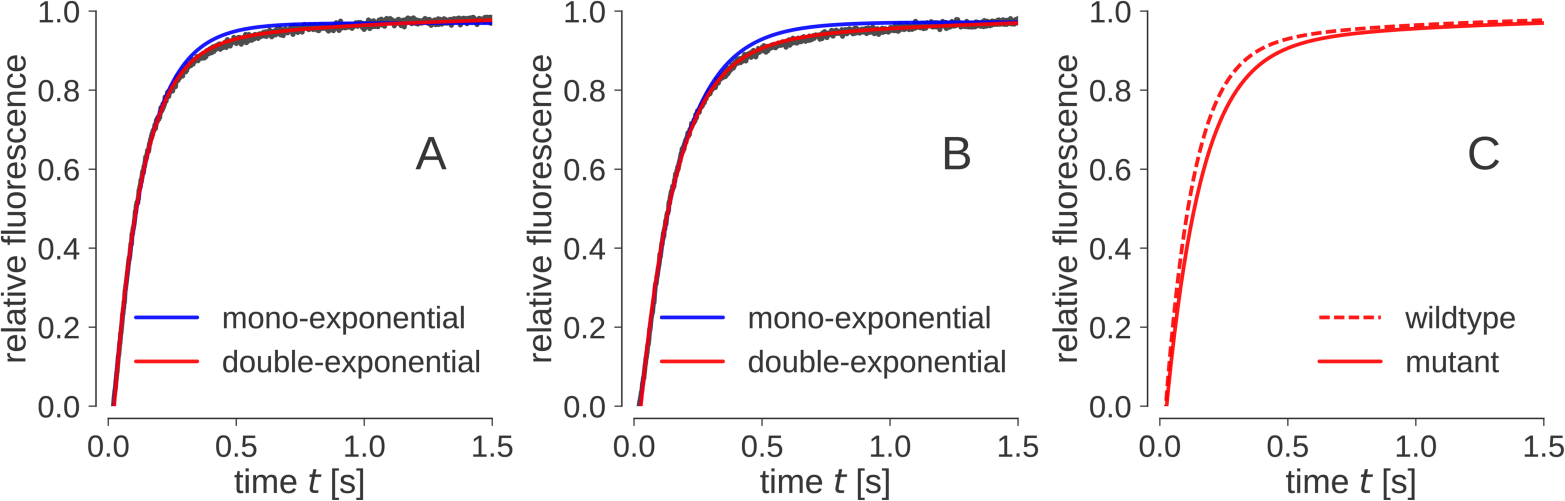
Raw stopped-flow data and exponential fits for wt (panel A) and Y80G OmpX (panel B). The raw data is shown in black, the single-exponential fit in blue, and the double-exponential fit in red. Clearly, a single exponential fit is not sufficient to describe the data. Panel C shows the difference between wt and Y80G OmpX double exponential fits (see also **Table 2**).

Rapid refolding was carried out by diluting the urea-unfolded OmpX into buffered detergent-rich solution (C8POE) in a stopped-flow setup. The raw data were subjected to multi-exponential fitting procedures using a self-written software that is based on a maximum likelihood approach (see methods section). The refolding trace was fit to single, double, and triple exponential functions to determine the best fit based on standard deviations and chi squared values (Table 2, and Fig 5). For the single exponential fit, a comparatively high chi-square value was found (Fig 5A, Fig 5B), while for the triple exponential fit the standard deviations were high compared to the double exponential fit. We therefore used a double exponential function to fit the refolding trace (Fig 5C). In wt OmpX, the first detected folding event is approximately 10-fold faster than the second folding event. When performing the same experiment with the Y80G mutant, we found the rate of folding to be significantly reduced compared to wt OmpX (Table 2). The first, fast step is reduced to 78% in Y80G OmpX compared to wt OmpX, while the second slower step is reduced to 60% of the rate observed for wt OmpX.

**Table 2:** Data from stopped-flow experiments of OmpX refolded into C8POE. Each individual stopped-flow measurement was fitted to a single, double and triple exponential function. The individual rates, amplitudes and chi-square values were then averaged and are shown here with standard deviations for wt and Y80G OmpX. The double exponential function consistently gave better results than single or higher exponential functions and the kinetic constants for the double-exponential fit are shown here (see also Methods section). For the estimation of wt OmpX and Y80G OmpX kinetic parameters, 429 and 438 single experiments were performed, respectively. The raw data of the experiments is provided in S1 File.

	wt OmpX	Y80G OmpX
Average rate 1 (in s^-1^)	8.95 ± 0.40	7.02 ± 0.19
Average amplitude 1	91.7 ± 0.68	91.7 ± 0.42
Average rate 2 (in s^-1^)	0.85 ± 0.24	0.51 ± 0.04
Average amplitude 2	8.3 ± 0.68	8.3 ± 0.42
Sample size	429	438
Avg. chi^2^	0,51 ± 0,16	1,67 ± 0,27

The initial folding of OmpX into detergent micelles from urea is likely to include at least three rapid events: Upon urea dilution, OmpX will collapse from an extended form, adsorb to detergent micelles, and simultaneously or shortly thereafter fold and insert β-strands into the detergent micelles. In the later folding stages, loops and a stable, hydrogen-bonded network will form. This final, slow folding event has been followed by NMR and HD exchange on the time-scale of seconds to hours in the detergents DPC and LDAO, but those experiments were not sensitive to the rapid initial folding events measured here [32]. Our stopped-flow experiments are sensitive to the rapid, initial folding events.

It is possible that the two events observed here are directly linked to the environments of the two tryptophan residues present in the structure, suggesting that the Y80G mutation affects different parts of the folding process in slightly different ways, as was observed previously for other membrane proteins with multiple Trp residues, e.g. with the help of single-tryptophan mutants [31]. In the folded state, W140 is located on the membrane- or detergent-exposed hydrophobic surface of the barrel of OmpX on β-strand 8, at the site where the barrel closes by interacting with strand 1, while W76 is located in a more water-accessible periplasmic turn. Y80 is in close proximity to W76 in both sequence and in space in both the folded and unfolded state [33,34]; based on this, we suggest that the faster folding component observed in our experiments is contributed mainly by W76 –as the structural changes between folded and unfolded state are minimal and thus potentially fast. The slower component would then mainly represent the slower process of barrel closure, where W140 is a sensitive indicator as it is located on strand 8 close to the C-terminus (see also Discussion section).

It is interesting to note that in NMR studies on unfolded OmpX in 8M urea, two regions of the protein retained residual structure mediated by a network on hydrogen bonds [34]. These two regions coincide with the location of the two Trp residues observed here: one is the periplasmic turn around W76 and intriguingly includes Tyr80 of the mortise-tenon motif; the other is the C-terminal region including W140. The C-terminal region is known to be essential for recognition by the Bam machinery *in vivo* [20,21], but we can only speculate if residual structure of the C-terminal motif is important for membrane insertion. Our *in vitro* folding data suggests that the periplasmic turn region around W76 is a folding core, in line with previous findings that this region is the only part of engineered, larger barrel structures that cannot be deleted [4]. Based on this, we suggest that the interaction between strand 5 and 6 mediated by the mortise-tenon motif of Y80 and G112 is indeed the initial event in 8-stranded barrel formation at least *in vitro*, after collapse of the region from an extended state. We speculate that the motif is specifically important for aligning the strands in the proper register, so that the barrel can close efficiently and without mismatch. The extreme evolutionary conservation of the mortise-tenon motif suggests that it plays an equally important role for initiation of folding events *in vivo*.

### Thermodynamics: Folding yield and thermal stability

**Fig 6:**
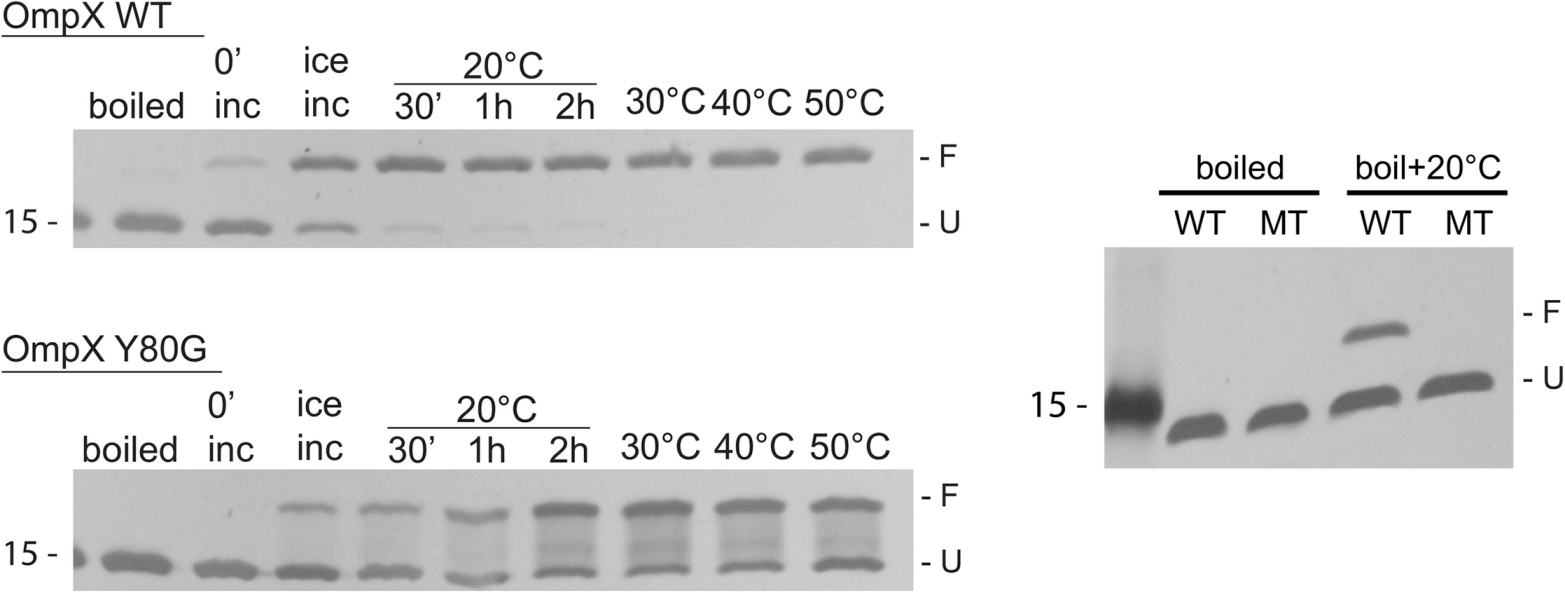
*In vitro* folding experiments for wt (WT) and Y80G OmpX (MT) at different temperatures. Protein samples in urea were diluted 1:20 into detergent-containing buffer, and incubated for 2h on ice, and at 20, 30, 40 and 50°C. The negative controls (boiled) in the gels are detergent-refolded samples which were boiled excessively at 100°C, while heated refolded samples (boil+20°C) are samples that after heating to 100°C for 1h to denature were shifted to 20°C for another hour to test for reversible refolding. The folded fraction (F) is represented by the upper band, while the unfolded fraction (U) is the lower band.

The *in vitro* folding efficiency for OMPs into lipid bilayers and detergents can vary with temperature [35,36]. In SDS sample buffer, folded OMPs retain their native state, unless they are heated (boiled) or treated with denaturants such as urea; they then migrate on an SDS-PAGE gel differently when compared to unfolded samples, an effect called a gel shift [37,38]; this can be used to assay folding yields and thermodynamic stability. Usually, smaller folded OMPs run at an increased molecular weight compared to their unfolded form, while for larger OMPs the effect is reversed [37,38]. OmpX, with eight β-strands, is considered a small OMP, and accordingly, the migration of the folded state appears at a higher molecular weight than that of the unfolded state [4]. For wt OmpX, the folding efficiency into micelles of the detergent SB-12 was found to be over 90% from ice-cold buffer to 50°C (Fig 6). In contrast, the Y80G mutant folded to only a negligible degree on ice (less than 5%, using identical buffer conditions), while increasing the refolding temperature to RT resulted in _~_50% folding efficiency (Fig 6, see also Materials and Methods). Despite the increased temperature necessary for folding, the similar gel shift for folded wt and Y80G OmpX does suggest that the final folded states of both wt and mutant are the same in terms of secondary and tertiary structure.

The difference in the total yield of properly folded wt vs Y80G OmpX supports the view that the presence of the mortise-tenon motif promotes a folding energy landscape where most protein molecules end up in a properly folded form, whereas the lack of this motif results in fewer protein molecules being rescued from pathways that can ultimately lead to misfolding. The observed increased efficiency in folding with the increase in temperature (for both wt and Y80G OmpX) can be explained by the activation energy required for OmpX folding into SB-12. Differences in the SB-12 micellar size and/or water-headgroup interactions at the different temperatures might also play a role. In general, detergent micelles become slightly smaller at increased temperatures, and water-head group interactions can also vary as a function of temperature [39–41]; we thus cannot exclude that temperature-dependent differences in SB-12 properties also affect OmpX folding efficiency.

We also performed a recovery experiment where following thermal denaturation of wt and Y80G OmpX at 100°C, the protein was incubated on ice to assess whether the protein could reversibly refold (Fig 6, gel 3). While _~_90% of wt OmpX was able to reversibly refold, OmpX Y80G was unable to refold under these conditions. While this to some extent may be an effect of the reduced hydrophobicity of the mutant, we believe that the recovery after denaturation, as well as the changed activation energy requirements for folding, are both more likely a consequence of the deleted mortise-tenon motif. This motif might act to thermodynamically stabilize transiently folded states by a zipper-like mechanism that brings together neighboring β-strands.

**Fig 7:**
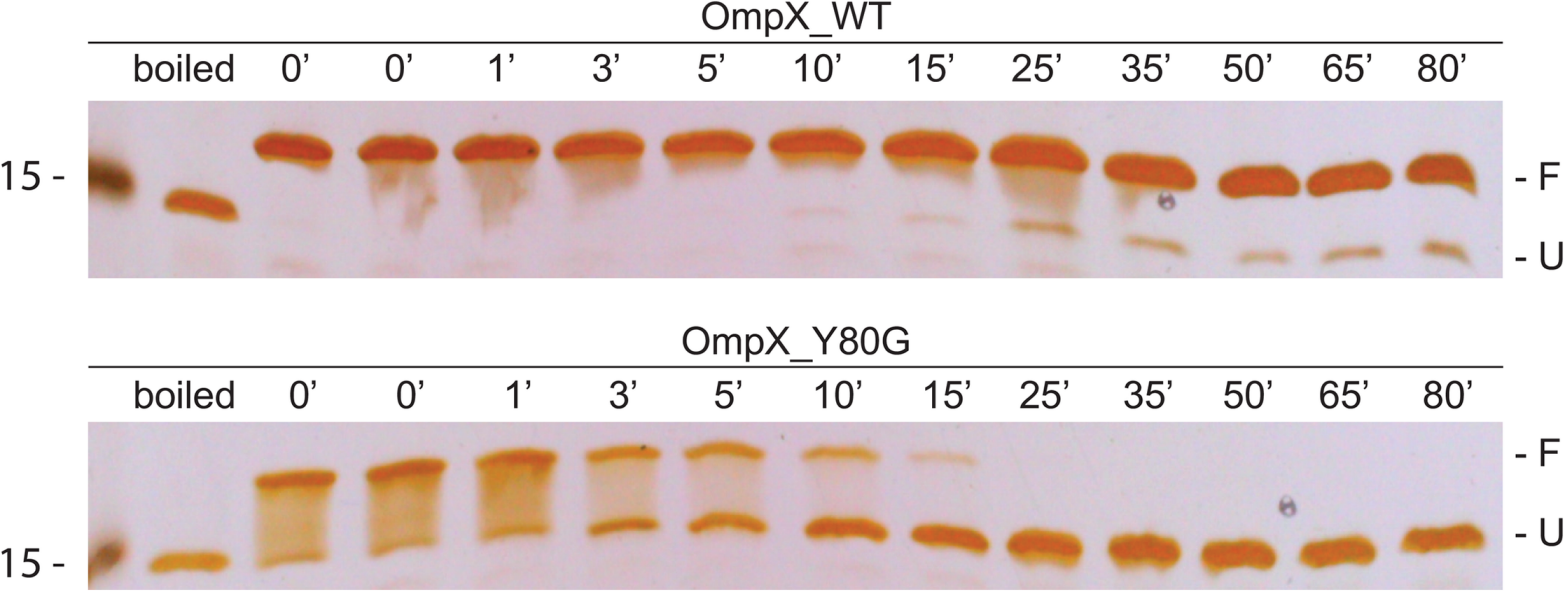
Heat stability for wt and Y80G OmpX monitored by gel shifts. Proteins were refolded *in vitro* prior to incubation at 85°C for the indicated time. The denatured OmpX appears as the lower band (U) on the gel, while folded OmpX is shifted to the upper band (F). An estimation of folded/unfolded yields of proteins can be made by comparing the intensities of the two bands. In the second lane of both gels OmpX samples were boiled at 100°C for 30 min before loading as a control.

To study the effect of the Y80G mutation on the thermal stability of folded OmpX, heat stability assays using SDS-PGE gels were carried out for both wt and Y80G OmpX (Fig 7). Refolded wt and Y80G OmpX were heated to 85°C for different time points, and then run on an SDS-PAGE gel to determine the levels of folded vs. unfolded protein (Fig 7). Y80G OmpX is clearly less thermostable than wt OmpX—while approximately 90% of wt OmpX is still folded after 80 minutes, Y80G OmpX is fully denatured after 25 minutes.

**Fig 8:**
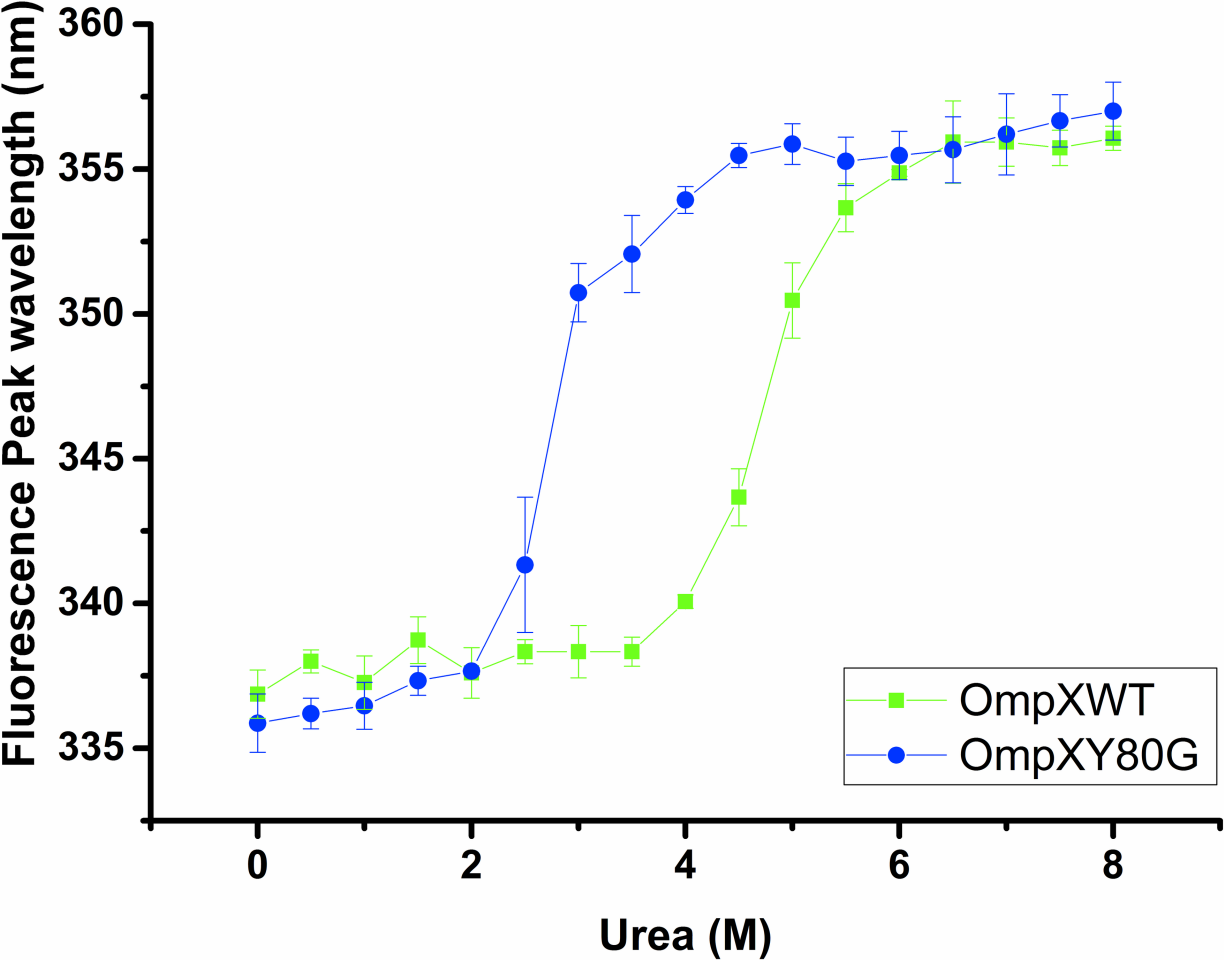
Stability of wt and Y80G OmpX as a function of urea concentration. As OmpX unfolds, the 2 tryptophan residues are exposed to a hydrophilic environment, resulting in a shift of the fluorescence maximum to higher wavelengths (336 nm to 356 nm), indicating that the protein has unfolded. Three technical replicates were used, and the averaged results are shown with standard deviation.

In addition, we have tested the stability of both wt and Y80G OmpX in the chaotropic reagent urea (Fig 8). Measuring the fluorescence spectra for both wt OmpX and Y80G mutant reveals that wt OmpX is stably folded in urea concentrations to up to 4M, while the Y80G mutant begins to denature at 2.5M urea.

Thus, along with the observed effects on protein folding kinetics, folding yield and refolding efficiency, the Y80G mutation also leads to decreased thermal stability of folded OmpX, and to decreased resistance towards chemical perturbations by chaotropic reagents. This is in accordance with studies on other OMPs where single point mutations can also stabilize the structure against the same type of perturbations [42]. We thus suggest that while Y80G OmpX maintains a native-like fold that is supported within detergent micelle, the final structure is nonetheless compromised, likely because strands 5 and 6 are not stably locked together, leading to an overall less stable secondary and tertiary fold.

## Discussion

In this study, we investigate the effect of a widely conserved mortise-tenon motif on the folding of beta-barrel proteins, and show that mutating the tyrosine of this motif in OmpX, leads to a change in folding kinetics, activation energy of folding (based on the requirement for higher temperature for refolding of the mutant compared to the wt protein, Fig 6), and stability of the folded state (Fig 7). Y80 in OmpX is present in a previously proposed hydrophobic cluster involving residues 73–82 of OmpX. This cluster was shown by NMR to interact transiently with DHPC micelles and to retain a non-random α-helical structure in 8M urea [33,34]. While W76 has been previously implicated to have a role in the formation and stability of this hydrophobic cluster, as well as in the kinetic stability of OmpX [43], this is the first time to our knowledge that the effect of mutating the tyrosine, and thus the mortise-tenon motif in this region, on OmpX folding has been studied.

Our study shows *in vitro* folding. *In vivo*, OmpX and other OMPs are bound to periplasmic chaperones prior to membrane insertion. In the structure of OmpX bound to the *E*. *coli* chaperone Skp, no specific regions of OmpX were recognized by Skp, and it was found that the protein was overall highly flexible. Compared to urea-unfolded OmpX, no residual secondary structure was present and the protein was found to be in a more compact state compared to urea-unfolded OmpX [44]. Importantly, the authors argued that the lack of specific interactions supports the idea that Skp is able to interact with a variety of β-barrel substrates. It has also been observed in the autotransporter Esp, that the mortise-tenon motif is not interacting either with Skp or BamA [45]. We would thus suggest that the role of Y80 in OmpX, and the corresponding mortise-tenon motifs that we describe for other OMP families, is not specific to chaperone-bound states *in vivo*, but stabilizes a conformational intermediate that increases the overall rate and efficiency of folding, and influences initial protein-lipid interactions that catalyze protein folding. The potential for protein-lipid interactions with Y80 is further supported by the observed hydrophobic interactions between DHPC micelles and the partially structured regions between residues 73–82 and residues 137–145 in urea-unfolded OmpX [33]. In the presence of the DHPC micelles, these two clusters of amino acids were found to have helical-like conformations with the hydrophobic side chains, including that of Y80, pointing outwards. The outward-facing conformation of the hydrophobic side chains would likely promote the observed interactions between these two regions and the DHPC micellar tails, which is supported by the fact that deletion of either W76 or W140 led to a loss in DHPC interactions with this two clusters, as well these region’s structural propensity.

**Fig 9:**
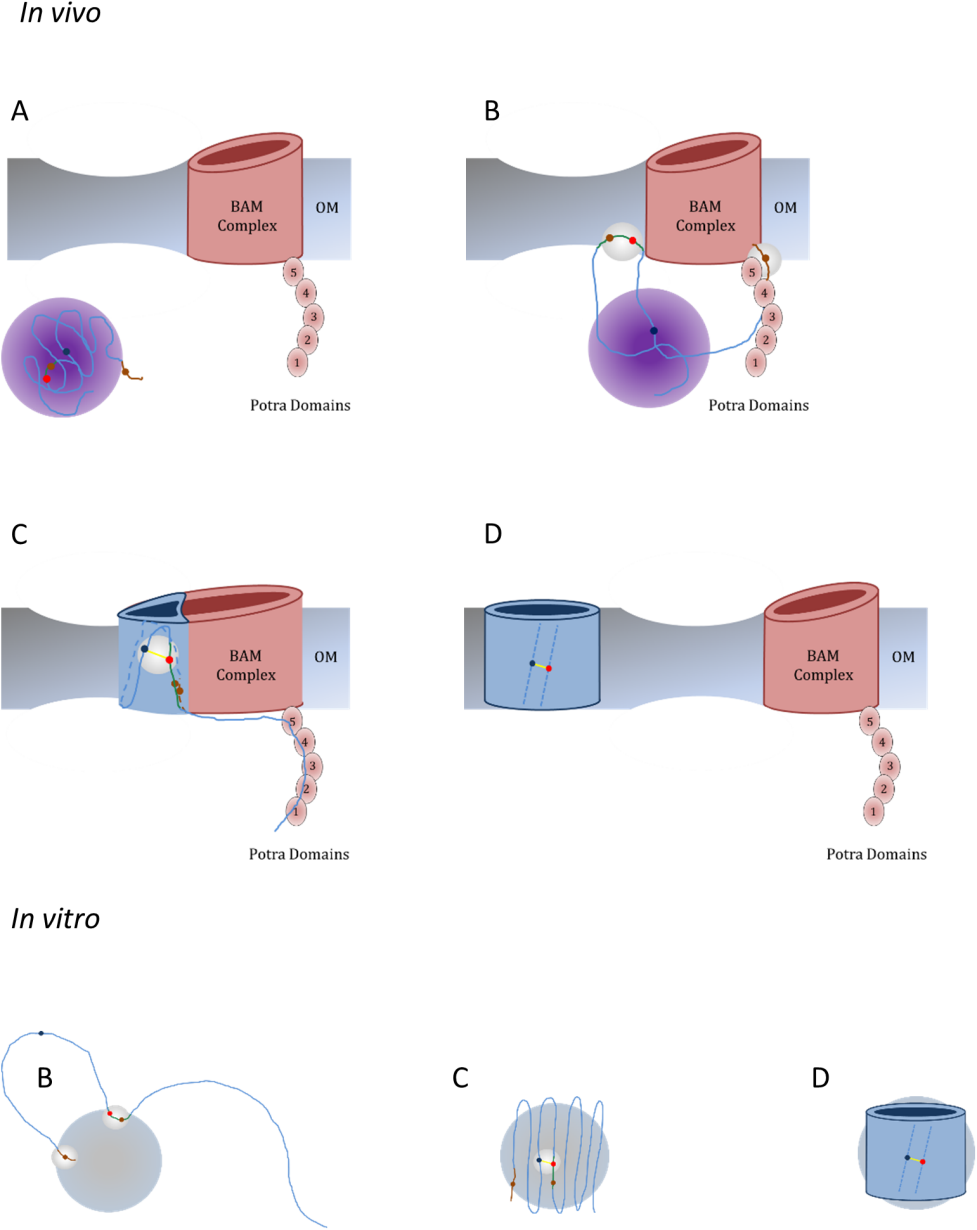
The folding of OmpX *in vivo* and *in vitro*. Highlighted in white spheres in both folding models are where similarities between the two folding processes may be present. ***In vivo***: The BAM and Skp-assisted folding of OmpX into the OM. **(A)** OmpX is held in a flexible, relatively packed state by the Skp complex. **(B)** Recognition of the β-signal by the BAM complex leads to its release from Skp, and delivery to the BAM Complex. Possible interactions between the periplasmic leaflet and the β -signal and region 73–82 may also occur. **(C)** The BAM-assisted folding and insertion of OmpX into the OM is stabilized by the G112-Y80 motif by aligning strands 5 and 6. **(D)** OmpX is released from the BAM complex, and the fully-folded protein is thermally stabilized by the G112-Y80 motif. ***In vitro***: **(B)** OmpX is expected to be extended in 8M urea compared to the Skp-bound form. Residual secondary structure and micelle interactions are present in the β-signal and region 73–82. **(C)** Upon urea dilution, OmpX will collapse and simultaneously fold into the DHPC micelle. This process is mediated by Y80G-Y112 motif, which kinetically increases the speed of folding by aligning strands 5 and 6. Strands 1 and 8 come together after folding of the protein core, followed by loop formation and a stable hydrogen bond network. **(D)** The final structure is thermally stabilized by the G112-Y80 motif.

Like for all enzymes, the role of BamA is to change a reaction pathway to remove kinetic barriers, rather than to change the thermodynamics of the reaction. Thus, while the expected mechanisms for the folding of OmpX *in vivo* vs. *in vitro* will be quite different in detail, we can assume that the start- and endpoints of the folding process are similar (Fig 9). Both *in vivo* and *in vitro*, it is likely that the G112-Y80 motif in OmpX plays a role in initiation of folding: by Y80 interacting with the membrane or micelle surface (panels B), by increasing the rate of protein folding (by helping to find the registered for the folding strands and hairpins, panels C), and/or by stabilizing the folded protein (by clamping neighboring strands together, panels D). We speculate that *in vivo*, this region and the C-terminal β-signal (which is essential for BAM recognition) [46] interact with the periplasmic leaflet of the OM upon release from Skp, similarly to the interactions observed between these regions and DHPC micelles.

**Fig 10:**
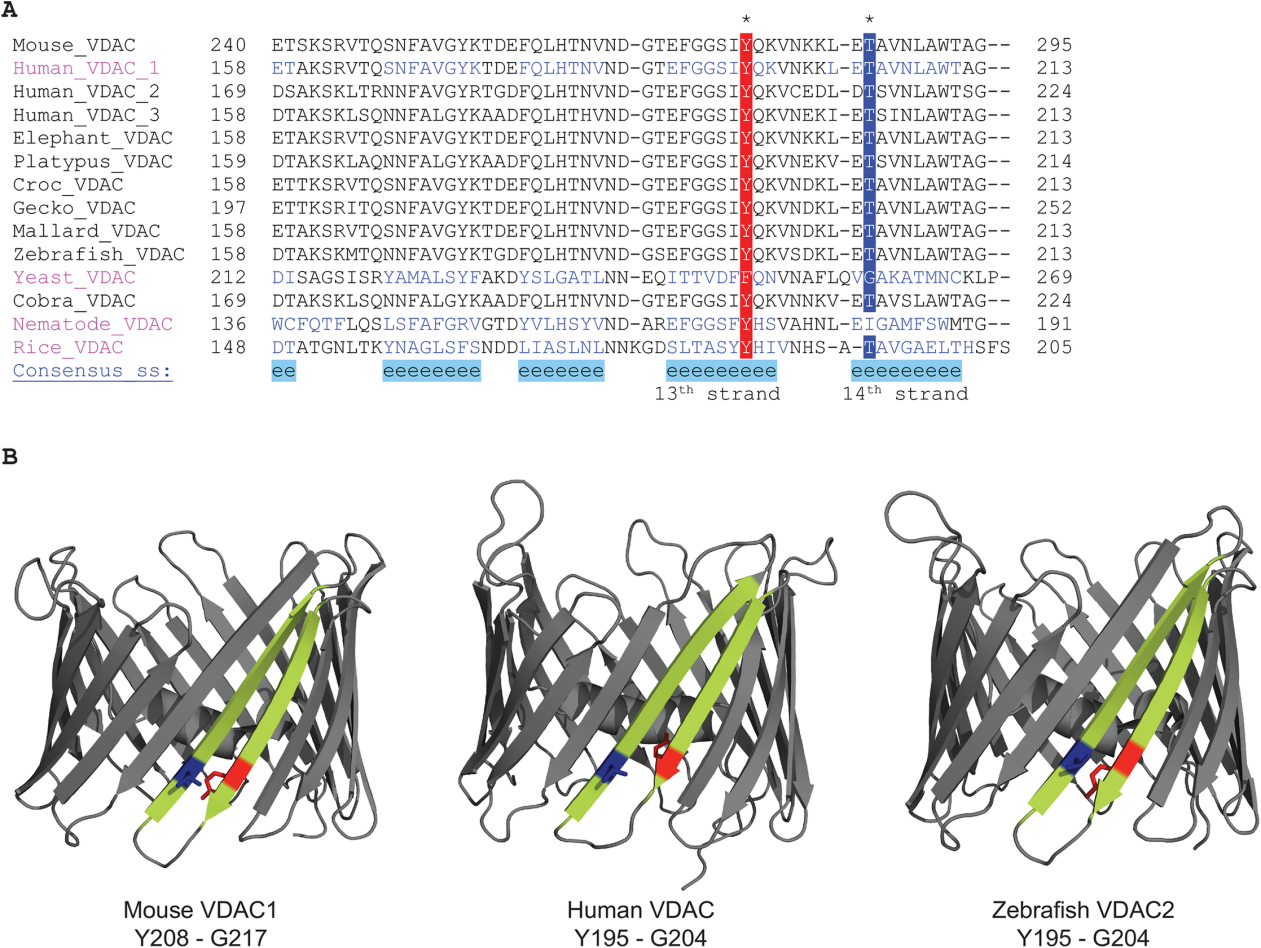
**(A)** Alignments of the mortise-tenon motif in VDAC proteins. The conserved motif is located on the 13^th^ and 14^th^ β-strands, and most commonly consists of a threonine and a tyrosine. The color scheme is the same as in Fig 2. **Mouse_VDAC—**AAB47777.1 isoform 1 [*Mus musculus*]; **Human_VDAC_1—**AAA61272 isoform 1 [*Homo sapiens*]; **Human_VDAC_2—**AAB59457.1 isoform 2 [*Homo sapiens*]; **Human VDAC_3**—AAB93872.1 isoform 3 [*Homo sapiens*]; **Elephant_VDAC—**XP_010588919.1 isoform 1 [*Loxodonta africana*]**; Platypus_VDAC**—XM_007668866.2 isoform 1 [*Ornithorhynchus anatinus*]**; Croc_VDAC—**XP_019390612.1 isoform 1 [*Crocodylus porosus*]; **Gecko_VDAC—**AAW79047.1. isoform 1 [*Gekko japonicus*]; **Mallard_VDAC**—XP_005011296.1 isoform 1 [*Anas platyrhynchos*]; **Zebrafish_VDAC**—AAH42329.1 isoform 2 [*Danio rerio*]; **Yeast_VDAC**—AAA35208.1 isoform 1 [*Saccharomyces cerevisiae* (strain ATCC 204508 / S288c)]; **Cobra_VDAC**—ETE66681.1 isoform 3 [*Ophiophagus hannah*]; **Nematode_VDAC**—CDP92897.1 isoform 1 [*Brugia malayi*]; **Rice_VDAC**—CAB82853.1—isoform 1 [*Oryza sativa* subsp. japonica]. **(B)** Available VDAC structures. The 13^th^ and 14^th^ β-strands containing the motif are highlighted in yellow with the marked mortise-tenon pair in blue and red respectively. The amino acid pair forming the motif is given under the names of the proteins. PDB codes: Mouse VDAC1 [3EMN]; Human VDAC1 [2JK4]; Zebrafish VDAC2 [4BUM].

To further access the conservation of this motif within non-bacterial β-barrels, we analyzed the protein sequence of the voltage dependent anion channel (VDAC). VDAC is an essential pore in the eukaryotic outer mitochondrial membrane that functions to transport substrates across the mitochondrial OM [47–50], and is the only eukaryotic β-barrel membrane protein whose structure is solved to date to high resolution, with structures of isoform 1 from both human and mouse [51–53], and isoform 2 from zebrafish [54]. All structures show the existence of the mortise-tenon motif on the 13^th^ and 14^th^ β-strands where the pair is formed most commonly between a threonine and tyrosine, in contrast to bacterial β-barrel OMPs where more commonly the pair is formed between a glycine and tyrosine (Fig 10, top panel). To further access the conservation of this motif, we aligned the sequences of VDAC channels from a diverse range of organisms, from yeast to plant to human, with sometimes very low pairwise sequence identity (less than 30%), and found near 100% conservation of this motif (Fig 10, upper panel). The high conservation of this motif in VDAC further supports the importance of this motif in all transmembrane β-barrels and may indeed be direct proof of an evolutionary link to prokaryotic β-barrels, which previous statistical analysis of sequence conservation [7] has not provided with high confidence.

All β-barrel OMPs seem to have evolved from a single, ancestral ββ hairpin through duplication events [7], which is further supported by the sequence similarity between individual β-hairpins [55]. Indeed, refolding studies with engineered OmpX variants have shown that by a simple duplication event, larger folded barrel structures can be formed in a single genetic operation. Intriguingly, constructs lacking a central hairpin containing β-strands 4 and 5, where Y80 is located on β-strand 5, were unable to fold in all variants tested, further supporting the idea of a central folding core within OmpX [4]. Conserved protein building blocks have been shown to exist throughout nature [56], and specifically within coiled coils there are local motifs that are required for triggering protein folding [57]. We propose that the mortise-tenon motif is important in folding and stabilizing one such conserved structural building block, a transmembrane ββ hairpin in β-barrel OMPs, with an additional important function in stabilizing folding intermediates and annealing neighboring strands in larger barrel structures. Extremely efficient insertion and folding of OMPs is required in fast-growing bacteria to avoid protein misfolding and subsequent negative effects such as inclusion body formation and the induction of the periplasmic stress response [58–60]. At the same time, the stability of folded proteins can be important in withstanding adverse environmental conditions such as elevated temperatures [14]. Together, both the kinetic and thermodynamic benefits of the mortise-tenon motif explain its extreme conservation in nearly all OMP families.

## Methods

### Bioinformatic analysis

To analyze the occurrence of mortise-tenon motifs in known OMP β-barrel structures, we used the SCOPe database, which groups them into 15 families within 7 superfamilies. To obtain multiple sequence alignments (MSA) for these families, we mapped them to their corresponding Pfam entries by performing sequence searches initiated with representatives of each family against the Pfam database[61]. The seed alignments provided for each family by Pfam were used for detecting conserved mortise-tenon motifs. Representative structures from each SCOPe family were analyzed manually to identify tenon-mortise motifs. Next, the respective multiple sequences alignments were analysed for the conservation of these motifs across the family; only motifs conserved in at least 70% of the sequences in the MSA were deemed important. For multiple structural alignments, the PROMALS3D webserver tool was used [62] with default settings. The NCBI accession numbers for the amino acid sequences used are provided in all relevant figure legends.

### Protein structure visualization

The opens source software PyMOL (https://www.pymol.org/) was used for 3D visualization of all structures.

### Cloning

WT *E*. *coli OmpX* without the signal sequence (residues 1–23) in pET3b (pET3bOmpX) was used from a previous study [4].

The tyrosine mutant (OmpX Y80G) was constructed using a two-step PCR mutagenesis protocol [63]. Briefly, the N- and C- terminal parts were amplified separately including a Tyrosine 80 to Glycine mutation in the primers

FP1: 5-GATTATCATATGGCGACTTCTACCGTAACTGGC

RP1: 5-GCCGATGCTTGCCCAGTCGTTAATGC

FP2: 5-GCATTAACGACTGGGCAAGCATCGGCGGTGTAGTGGGTGTGGGTTATG

RP2: 5-AAAAGAGGATCCTTAGAAGCGGTAACCAACACCG

The two resulting PCR products were fused in a second PCR via their complementary sequences using the forward and reverse primers from the first step, and the product was digested and cloned into pET3b using the NdeI and BamHI sites as above (pET3bOmpX_Y80G). All constructs were transformed into chemically competent *E*. *coli* TOP10 cells (Invitrogen), and confirmed by sequencing.

### Protein production and purification

Large-scale protein expression and purification was achieved using previously described methods [4]. In brief, pET3bOmpX and pET3bOmpX_Y80G were transformed into chemically competent *E*. *coli* C41(DE3) cells. Cells were grown at 37°C in LB medium in shaking flasks, induced with 1mM IPTG (isopropyl-β-d-thiogalactopyranoside) when the OD600 reached 0.6, and harvested after 4h by centrifugation at 4°C.

Cells containing inclusion bodies were suspended in PBS supplemented with 10mM MgCl_2_, 10 mM MnCl_2_ and a pinch of DNase, and lysed with a French Press three times. Inclusion bodies were purified from the membrane debris by slow centrifugation for 15 min at 2000g. The resulting pellet was resuspended in 1,5% (v/v) Triton X-100 in PBS, centrifuged again at 2000g, followed by washing with MiliQ water three times to remove the detergent. The crudely purified inclusion bodies were dissolved in urea buffer (6M Urea, 1mM EDTA, 20mM Tris-HCl pH8.5) and purified using a GE Healthcare FPLC system using a 20 ml Mono Q anion-exchange column with a linear gradient of 0–1 M NaCl in urea buffer. The protein purity was checked on an SDS-PAGE gel. Samples were diluted to the desired concentration in urea-containing buffer.

### Stopped-flow spectroscopy

Kinetic refolding traces were obtained using a BioLogic stopped-flow apparatus SFM-300/s—MOS-200 with a FC-08 microcuvette accessory. All experiments were done at 25°C. The excitation light was set to 295 nm and the emission was recorded with a cut-off filter at 320 nm. One 2mL syringe was loaded with unfolded protein at 5 mg/ml concentration in urea buffer (6M Urea, Tris-HCl pH8.5, 1mM EDTA), and the two remaining 10 mL syringes were loaded with detergent-rich buffer (1% C_8_POE (w/v) (octyl-polyoxyethylene), Tris-HCl pH8.5, 1mM EDTA). The mixing ratio was set to 1:20 (v/v protein to detergent solution), and a flow of 14 mL/s was applied at a final volume of 422 μl per experiment. The acquisition start time was set to 10ms before the stop of mixing to minimize the dead time. Due to software limitations for the number of time points that could be recorded, we divided the acquisition into 3 segments: timebase 1 with 5001 data points recorded every 0.00005s; timebase 2 with 2749 data points recorded every 0.001s, and timebase 3 with 44 data points recorded every 0.5s. 500 single shots were collected for both wt and Y80G OmpX, and the raw data analyzed by custom-designed kinetic analysis software described in the following section.

### Kinetic analysis (software)

Stopped-flow data were analyzed with in-house software developed by us. Our method is based on a maximum likelihood approach that fits multi-exponential decays directly to the kinetic refolding traces. This is based on the following standard equation for multi-exponential fits,

$$f(t)=c+b\;t-\sum_{x=1}^{n}a_{x}e^{-k_{x}t}$$

where c is the offset (constant baseline) of the measurement, b is an optional linear component that corrects for instrument drift and that was minimal for our experiments, a is the amplitude and k the rate constant of each exponential component. Thus, each exponential decay has two parameters, an amplitude and a rate constant. Therefore, the total number of parameters that needs to be fitted is twice the number of exponentials plus an additional parameter for the baseline. We account for deviations from the ideal model by assuming a Gaussian noise model whose parameters we also estimate (for each of the three time bases we estimate a separate noise level). The maximum likelihood approach results in a non-linear least-squares fitting problem that we solve by running the Nelder-Mead algorithm starting from randomly varying initial conditions. Additional exponential components are added progressively, starting from the best fit obtained with the previous model and adding one additional exponential component one by one. We fit each refolding trace in a fully automated way starting with a single exponential decay, which is successively refined to a maximum of five exponential decays. By monitoring the chi-squared residual as a function of the number of exponentials, we find that both the wt as well as the mutant data can be modelled with two exponentials; higher order models do not improve the quality of the fit significantly. Our software is implemented in Python 2.7 and depends on the numpy and scipy libraries (Habeck et al. In preparation).

For comparison, the single-, double, and triple-exponential fit parameters are shown in Table 3.

**Table 3:** Fit parameters for different exponential fits. The chi-square values suggest that a single-exponential fit does not describe the data well, while the standard deviations of the individual rates increase dramatically for the triple-exponential fit, to a level where the errors are as large as the calculated values for rates (and amplitudes) 2 and 3.

	single exponential fit		double exponential fit		triple exponential fit	
	wt	Y80G	wt	Y80G	wt	Y80G
Avg. rate 1	8,03 ± 0,3	6,45 ± 0,17	8,95 ± 0,40	7,02 ± 0,19	9,07 ± 0,45	7,02 ± 0,18
Avg. amp. 1	100 ± 0	100 ± 0	91,7 ± 0,68	91,7 ± 0,42	85,68 ± 9,14	87,80 ± 10,62
Avg. rate 2	-	-	0,85 ± 0,24	0,51 ± 0,04	2,44 ± 2,83	1,26 ± 2,07
Avg. amp. 2	-	-	8,3 ± 0,68	8,3 ± 0,42	10,34 ± 7,41	10,87 ± 8,27
Avg. rate 3	-	-	-	-	3,61 ± 2,97	3,41 ± 1,96
Avg. amp. 3	-	-	-	-	3,97 ± 3,21	1,33 ± 2,74
Avg. chi^2^	2,52 ± 0,59	3,41 ± 0,69	0,51 ± 0,16	1,67 ± 0,27	0,53 ± 0,25	1,19 ± 0,34

The software is provided in S2 File.

### Folding, refolding and heat stability

The folding, refolding and heat stability for both wt and Y80G OmpX was accessed *in vitro* in the detergent SB-12 (N-Dodecyl-N,N-dimethyl-3-ammonio-1-propanesulfonate. For the folding experiment, the folding reaction was carried out for 2 hours on ice, and at 20, 30, 40 and 50°C. SDS page loading buffer was added to stop the refolding reaction, and the samples were directly loaded onto SDS page gels without centrifugation or heating. For refolding experiments, wt and Y80G were denatured at 100°C followed by 2 hours of incubation at 20°C prior to loading on an SDS page gel. For the heat stability assays, Denatured protein in 8M Urea was firstly refolded by rapidly diluting it 1:20 into pre-chilled buffer with SB-12, followed by RT incubation for 2 hours. The samples were centrifuged to remove aggregated proteins and transferred to a water bath heated to 85°C for defined time periods. Samples were removed from the water bath at the indicated time points, and SDS-PAGE loading buffer was added immediately to arrest the reaction [4]. All samples were loaded onto a 15% SDS-PAGE in a water-cooled chamber to keep the temperature low, and silver-staining method was used for detection [64]. Estimation of folding yields were based on band intensities.

### Fluorescence measurements of urea-unfolded samples

Fluorescence measurements were essentially performed as described earlier [4]. In brief, samples of folded OmpX were mixed with detergent buffer and urea stock solutions to achieve constant protein and detergent concentrations at different concentrations of chaotrope as indicated in Fig 8. Measurements were taken with a JASCO FP-8000 series spectrophotometer using standard settings, with an excitation wavelength of 280 nm.

## Supporting information

S1 File (ZIP)

S2 File (ZIP)
